# Simulation of facial growth based on longitudinal data: Age progression and age regression between 7 and 17 years of age using 3D surface data

**DOI:** 10.1371/journal.pone.0212618

**Published:** 2019-02-22

**Authors:** Jana Koudelová, Eva Hoffmannová, Ján Dupej, Jana Velemínská

**Affiliations:** 1 Department of Anthropology and Human Genetics, Faculty of Science, Charles University, Prague, Czech Republic; 2 Department of Software and Computer Science, Faculty of Mathematics and Physics, Charles University, Prague, Czech Republic; universita’ degli studi di milano, ITALY

## Abstract

Modelling of the development of facial morphology during childhood and adolescence is highly useful in forensic and biomedical practice. However, most studies in this area fail to capture the essence of the face as a three-dimensional structure. The main aims of our present study were (1) to construct ageing trajectories for the female and male face between 7 and 17 years of age and (2) to propose a three-dimensional age progression (age -regression) system focused on real growth-related facial changes. Our approach was based on an assessment of a total of 522 three-dimensional (3D) facial scans of Czech children (39 boys, 48 girls) that were longitudinally studied between the ages of 7 to 12 and 12 to 17 years. Facial surface scans were obtained using a Vectra-3D scanner and evaluated using geometric morphometric methods (CPD-DCA, PCA, Hotelling’s T^2^ tests). We observed very similar growth rates between 7 and 10 years in both sexes, followed by an increase in growth velocity in both sexes, with maxima between 11 and 12 years in girls and 11 to 13 years in boys, which are connected with the different timing of the onset of puberty. Based on these partly different ageing trajectories for girls and boys, we simulated the effects of age progression (age regression) on facial scans. In girls, the mean error was 1.81 mm at 12 years and 1.7 mm at 17 years. In boys, the prediction system was slightly less successful: 2.0 mm at 12 years and 1.94 mm at 17 years. The areas with the greatest deviations between predicted and real facial morphology were not important for facial recognition. Changes of body mass index percentiles in children throughout the observation period had no significant influence on the accuracy of the age progression models for both sexes.

## Introduction

The human face is a complex biological structure with fixed anatomical features (e.g. eyes, nose, lips, chin). The spatial position and relative proportions of each facial component constitute some of our most distinguishing visible traits. These individual traits, together with features that are characteristic of specific groups defined by age, sex or ancestry affiliation, are essential for the identification of individual persons [1,2]. Some of the most difficult tasks in forensic practice are connected with human age, especially with facial age estimation and age progression. The need for accurate and objective methods for identifying individuals is related to current socio-political developments. The reasons include an increasing number of cases requiring age estimation in living individuals with no valid proof of date of birth and a large number of missing persons. The identification of such subjects may clarify cases with significant legal and social ramifications for individuals as well as for society [3].

According to the International Commission on Missing Persons (ICMP) [4], thousands of persons go missing every year as a result of war conflicts, human rights violations, disasters, organized violence, or refugee flows and migration. The International Organization for Migration (IOM) [5] estimates that one billion people—out of the seven billion people on the planet—are on the move as migrants. A large proportion of the cases are missing children younger than 18 years. According to the National Centre for Missing and Exploited Children (NCMEC) [6], approximately 800,000 children are reported missing in the USA every year. Missing Children Europe (MCE) [7], the European federation for missing and sexually exploited children, reported 250,000 children missing every year in the EU, and, for example, that only 46% of the children reported missing in 2015 were found within the same year. According to Europol [8], at least 10,000 refugee children are unaccounted for after arriving in Europe, with many feared to be exploited and abused for sexual or labour purposes. However, national reports seem to suggest that the number of missing unaccompanied children could be much higher and that many children go missing even before being registered by the authorities. According to Lampinen et al. [9], in more than 50% of cases the child has been missing for more than three years, and in a quarter of the cases the child has been missing for more than a decade. Resolving cases where a child is missing for that length of time is complicated by the fact that children change dramatically as they age, making their identification difficult [10].

The age progression techniques routinely employed to assist in the location of missing persons currently rely on the skills of trained forensic artists able to age a face without the direct employment of scientific principles [11]. Most forensic artists use photographs of biological relatives (e.g. parents, siblings, grandparents) to improve the prediction of the current facial appearance [12,13]. In recent years, however, a number of face ageing systems have been proposed. There are two main approaches to studying age progression: prototyping-based age progression and modelling-based age progression [14]. Both these approaches require face ageing sequences for the same person spanning a wide range of ages, which are very costly to collect [15]. Most authors have used face ageing datasets consisting of photographs, for example AGFW, CACD, FG-Net or MORPH [14–17]. Knowledge of age-stable features (i.e. moles, diameter of the iris) is another factor that improves age progression images [18,19]. Nevertheless, the they are difficult to get right, subjective and variable even when a high degree of anatomical and artistic modelling expertise is applied [20].

Progress in computer science and improvements to medical imaging technologies have led to the development of computer systems for forensic facial reconstruction [21,22] or for three-dimensional modelling of facial ageing using longitudinal [23,24] and cross-sectional data [25].

Successful facial classification and recognition of individuals according to their age requires knowledge about ontogenetic development [26]. Facial morphology changes with time and does not progress at a uniform pace [27]. For example, puberty causes significant changes to the face over a short period of time [28]. Facial shape changes associated with postnatal ontogenetic allometry are very similar among diverse regional groups, but subtle differences are present. Developmental shape changes are less consistent in early ontogeny compared to later ontogeny, during which developmental shape changes are very similar among different regional groups [29]. Nevertheless, the specification of population-specific ontogenetic trajectories is required.

The proportion of body fat is another factor that influences facial morphology and plays an important role in facial shape variation of adolescents as well as adults. Generally, relative widening of the mid- and lower face, reduction of eye height, widening of the nose, thickening of the lips and down-turning of the corners of the mouth occur with increasing body fat [30–32]. The optimal tool for measuring changes in adiposity during childhood is the body mass index (BMI), either by itself or as the proportional (percentage) change in BMI, which can be adjusted for sex and age [33].

Our previous study [23], which dealt with longitudinal facial growth of juveniles aged between 12 and 15 years, was focused on ontogenetic trajectories and proposed an age progression model. In the present study we also evaluated facial morphology but widened the age interval to span from childhood to adolescence (from 7 to 17 years of age). We supposed that the widening of the interval to earlier and later stages of facial development would help to identify certain ontogenetic traits typical for this period (i.e. the onset of puberty), which could improve the age progression model. From a legal perspective, the interval included the age of criminal liability (i.e. 15 years of age in Czech Republic). In the current study we also tested an age regression model that could be helpful in comparing images of younger and older children to determine whether they are the same individuals. Using a 3D stereophotogrammetric scanning system in conjunction with modern geometric morphometric methods, we attempted to simulate age-progressed effects on facial scans for both sexes based on the following partial aims: (1) to confirm facial age changes (the effect of age) between particular categories (from 7 to 17 years of age) within each sex, (2) to evaluate facial ageing trajectories separately for boys and girls over the whole observation period, (3) to propose an age progression (age regression) model and describe age-related facial changes, and (4) to test the effect of changes in body mass index (BMI) percentiles over the observation period on the age progression (regression) model.

## Subjects and methods

The study was approved by the Institutional Review Board of Charles University, Faculty of Science on September 2011. The subjects were children from a high school in the town of Kladno and an elementary school in Prague, Czech Republic, who were longitudinally studied between 2009 and 2015. The complete study sample consisted of two longitudinal sets of 3D facial surface scans from 7 and 17 years of age (the first group consisted of 23 girls and 17 boys scanned once a year between 7 to 12 years of age; the second one consisted of 25 girls and 22 boys scanned once a year between 12 and 17 years of age, too). The parents of all the children were informed about the 3D optical-scanning procedures and had expressed their consent with the investigation. The inclusion criteria of the study were: Caucasian origin, absence of craniofacial anomalies and facial trauma. The height and weight of each child were measured every year. BMI was calculated as body weight (in kilograms)/body height^2, (in metres^2). The percentile BMI graph can be used to accurately evaluate individuals’ BMI within the range of variability of this index for the population of corresponding age. For the construction of BMI percentile curves for the Czech population, the LMS method was used [34], based on the Box-Cox power transformation. In the Czech Republic, subjects with BMI values between the 25th and the 75th percentile are considered to have normal weight. Values between the 75th and the 90th percentile signal overweight children, and the 90th percentile is the upper cut-off threshold for identifying overweight children. Children whose BMI-for-age values are above the 97th percentile are identified as obese. Those with BMI values below the 25th percentile have a reduced body weight [35,36]. All statistical processing was performed in R 3.3.2. Significance was decided at the level of α = 0.05.

### Scanning and image processing

Acquisition of three-dimensional surface models was performed in two stages. First, each subject’s face was captured using a structured light-based optical scanner Vectra 3D (Canfield Scientific Inc., Fairfield, NJ). The subjects were scanned sitting on a chair and asked to make a neutral facial expression. The bundled software, Mirror PhotoTools (Canfield Scientific Inc., Fairfield, NJ), was used to control the scanner and export high-resolution surface models.

Next, these surface models were manually processed in RapidForm 2006 (INUS Technology Inc., Seoul, South Korea). Manual removal of extraneous data (parts of the neck, ears and hair) was carried out by a trained anthropologist. Finally, each surface was simplified to approx. 25k vertices using the programme’s inbuilt tools.

### Homology of surface representations

Homology of representation is crucial for any study in geometric morphometry. In triangle meshes, such as those used in this study, homology translates to vertices with the same index, describing the same anatomical feature. Unfortunately, surface representations generated by neither the scanner nor RapidForm, exhibit the said property. We therefore used the proven workflow CPD-DCA (coherent point drift—dense correspondence analysis) [37] to create homologous representations of our facial surfaces.

CPD-DCA works in stages. The first stage requires a small set of landmarks placed on each of the surfaces under study. We used a set of nine landmarks at the following locations: exoR = right exocanthion, exoL = left exocanthion, enR = right endocanthion, enL = left endocanthion, N = nasion, Pn = pronasale, chR = right cheilion, chL = left cheilion and Pg = pogonion. These landmarks were used for rigid pre-alignment only, which accelerated convergence in the next stage.

For the next stage, a template surface (also known as the base mesh) had to be selected arbitrarily. An automatic non-rigid registration algorithm was used to fit that base mesh on to each other surface (a technique known as floating surfaces). That fitting was then made tighter by projecting the registered vertices of the base mesh on to the floating surface. This produced a representation of the floating mesh, which was homologous to the base mesh.

Vertices that had been improperly matched in the previous stage were identified and removed from all specimens in the sample. Finally, generalized Procrustes analysis (GPA) was used to rigidly align the homologous surface representations. The resulting vertices in these aligned surfaces are also known as quasi-landmarks and can be treated as ordinary landmarks.

Because of the still high number of quasi-landmarks (about 25k per specimen), dimension reduction was performed using principal component analysis (PCA). This entire workflow is implemented in Morphome3cs [38].

### Visualization of ageing patterns

We constructed a progression of heat maps to visualize average year-to-year changes exhibited by the faces under study. Thanks to the vertex homology that we created in the previous step, it was possible to express the year of facial growth of a particular specimen by subtracting the respective vertex coordinates from two specific measurements. To construct a visualization of the transition from a specific age to the next year, we averaged these vertex coordinate differences, motion vectors as it were, from all series (subjects) that were scanned at both these ages. The visualization of each transition consists of two colour maps. One visualizes the length of the said mean motion vectors. The other map shows the statistical significance at each vertex. It is constructed by running Hotelling’s T^2^ tests on the motion vectors for a particular vertex from all available specimens and mapping the resulting p-values to the vertices. If a particular vertex exhibited a p-value of less than 0.05, we deemed its location as undergoing significant ageing-related motion.

### Facial averaging

We constructed a separate ageing trajectory for each sex. In the following paragraphs we describe the creation of the ageing trajectory for one of the sexes; the other is constructed analogously. Our data consisted of two longitudinal datasets; one from 7 years to 12 and the other from 12 to 17 years of age. The ageing trajectory was created by modelling principal component scores.

We discovered that there is a considerable amount of noise in the ageing progression of individual specimens. We therefore chose to simplify the ageing model in each longitudinal group to a linear one. Specifically, we fitted a linear model to each series in a particular group and obtained the group model by calculating the mean intercept and slope of the individual models. This gave us two partial trajectories: one for the 7–12 years old group and one for 12–17-year-olds. As the final step, we glued these two trajectories with a Hermitian cubic function that smoothly interpolated between their centres.

For each sex, we constructed a plot which visualized individual ageing models and a global compound model consisting of per-category mean models and a cubic interpolation.

### Prediction using the fitted model

The model obtained in the previous subsection can be applied in the space of principal component scores. We simulated ageing by transforming a specimen into principal component space and adding the desired portion of the ageing trajectory. The resulting principal component scores were transformed back to facial surfaces. Note that this model can be used not only for forward prediction, as we have done, but also retrospectively.

We measured the magnitude of the error by comparing our predicted faces against the ground truth. In each age group we took the youngest occurrence of each specimen and simulated their ageing to the oldest known occurrence. That prediction was compared against the known face by calculating distances between respective vertices, which was facilitated by vertex homology. These prediction errors were averaged from all specimens in a group and plotted as a colour map.

To determine whether our age progression framework provided useful data, we calculated prediction error values for each specimen using the ageing algorithm and using no prediction at all, using a non-aged face for the estimation. We compared the errors using a paired t-test to ascertain if there was a systematic improvement of the prediction.

### Prediction error due to BMI

We further examined the relationship between the mean prediction error and the severity of obesity. As a measure of obesity we used the percentile of BMI within each age and sex group, instead of raw BMI. The percentile of BMI was calculated using the software Rust.cz, which outputs the BMI percentile for a given sex, age, weight and height based on a large study performed on the Czech population [39]. We constructed linear models of the prediction error by BMI percentile and determined its significance using ANOVA for linear models.

## Results

**Fig 1:**
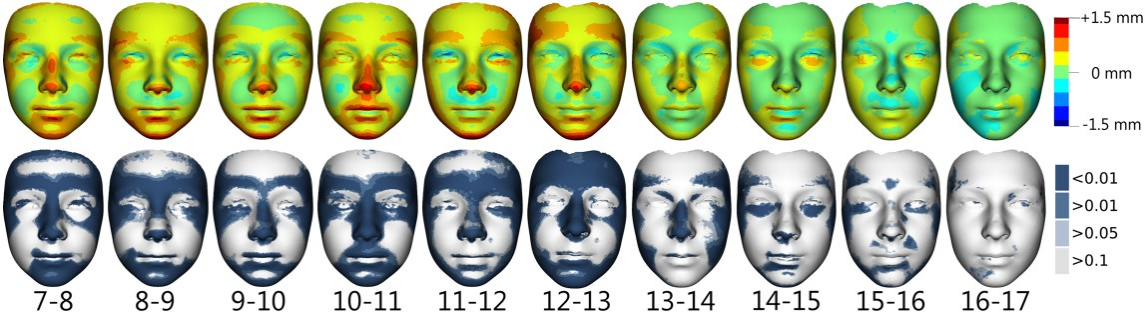
Visualization of facial growth changes between the ages of 7 and 17 years in girls. Shell distances of superimposed average facial forms of particular age categories are represented by colour deviation maps supplemented with colour histograms (upper row). The most protrusive parts of the average faces are represented in red whereas those which are situated deeper are coloured blue. The statistical significance of form differences was analysed per vertex and coded in blue shades (significant differences) or grey (no significant difference) on the superimposed average faces (lower row).

Superimposition of average faces between each age category was used to evaluate growth changes in facial morphology in 7 to 17 years old girls and boys. In girls (Fig 1) the most evident growth changes occurred between 7 and 13 years of age. Generally, an overall elongation of the face was observed between each age category up to the age of 13 years, especially in terms of the lengthening of the forehead and the lower face. An increasing prominence of the eyebrow ridges, nose tip and chin was observed from 7 to 14 years of age, with the greatest accentuation between the ages of 10 and 14 years; after this age, no marked changes in these parts were apparent. In the area of the cheeks (mostly their lateral parts), noticeable growth changes were apparent up until the age of 14 years. The region around the eyes became deeper in relation to the facial plane, except in the age categories from 14 to 16 years, where anterior growth of the region under the eyes occurred. As for the lips area, marked changes (anterior growth of both lips) were evident from 7 to 11 years of age. A deepening of the mentolabial crease was observed up to the age of 13 years, especially in relation to the more prominent lower lip and chin. Overall, the most significant growth changes occurred between the ages of 12 and 13 years—growth of the whole forehead region, the nose region and the mandible. After the 13th year of age, the growth changes slowed down. Between the ages of 14 years and 16 years, no or minimal changes (anterior changes of lower eyelids and the mentolabial crease) occurred, and after the age of 16 years, growth practically stopped. These results are apparent from both the colour-coded maps and the statistical significance maps.

**Fig 2:**
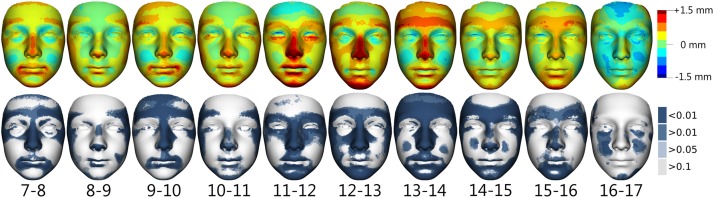
Visualization of facial growth changes between the ages of 7 and 17 years in boys. Shell distances of superimposed average facial forms of particular age categories are represented by colour deviation maps supplemented with colour histogram (upper row). The most protrusive parts of the average faces are represented in red whereas those which are situated deeper are coloured blue. The statistical significance of form differences was analysed per vertex and coded in blue shades (significant differences) or grey (no significant differences) on the superimposed average faces (lower row).

In boys (Fig 2) between 7 and 11 years of age, slight growth of the forehead, the nose, the lips area and the chin was observed. The annual growth increases in these facial regions remained rather steady. The most noticeable growth changes occurred after 11 years of age and continued until the age of 14, especially in terms of anterior growth of the forehead and eyebrow ridges, increase of nose prominence and enlargement of the downward projection of the chin. In addition, a flattening of the cheeks (especially between 13 and 15 years) and deepening of the orbit region in relation to more prominent eyebrow ridges occurred in all age categories and became more marked with age. After 14 years of age, facial growth slowly decreased, but still persisted up until the end of the observation period. After 16 years, growth changes were observed only in a minor part of the nose and chin.

**Fig 3:**
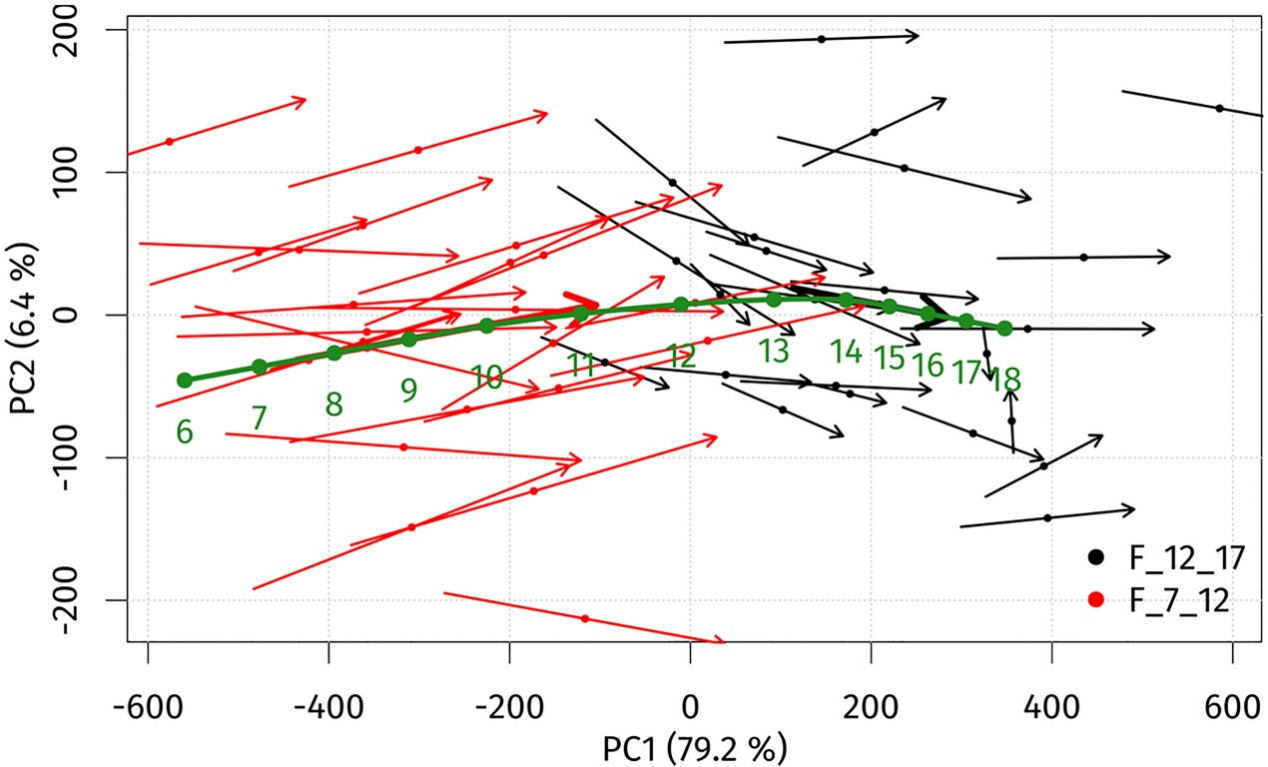
PCA scatter plot histogram for individual ageing trajectories (thin arrows), average ageing trajectories (thick arrows) and the global ageing trajectory (green arrow) of girls from 7 to 17 years of age in the space of the first (PC1) and second (PC2) principal components.

The ageing trajectories for girls and boys were constructed in the principal components (PC1, PC2) space to observe the ageing trends between the ages 7 and 17 years. A PCA scatter plot with ageing trajectories for girls is shown in Fig 3. The first two principal components accounted for over 85.6% of the sample’s variability. The first principal component (PC1) explained 79.2% of total variability and represents the facial ageing process. Towards positive values of PC1, the age of individuals increased. PC2 explained 6.4% of total variability and was associated with changes affected by facial concavity/convexity. Individual ageing trajectories of girls between 7 and 12 years were longer than in 12–17-year-olds, indicating different growth rates, which are better documented by the global ageing trajectory. The global ageing trajectory indicated steady annual growth changes up to the age of 10 years. The most intensive and pronounced facial growth changes occurred between 10 and 14 years of age and were much more pronounced between 11 and 12 years. After 14 years of age, annual growth changes slowed down rapidly.

**Fig 4:**
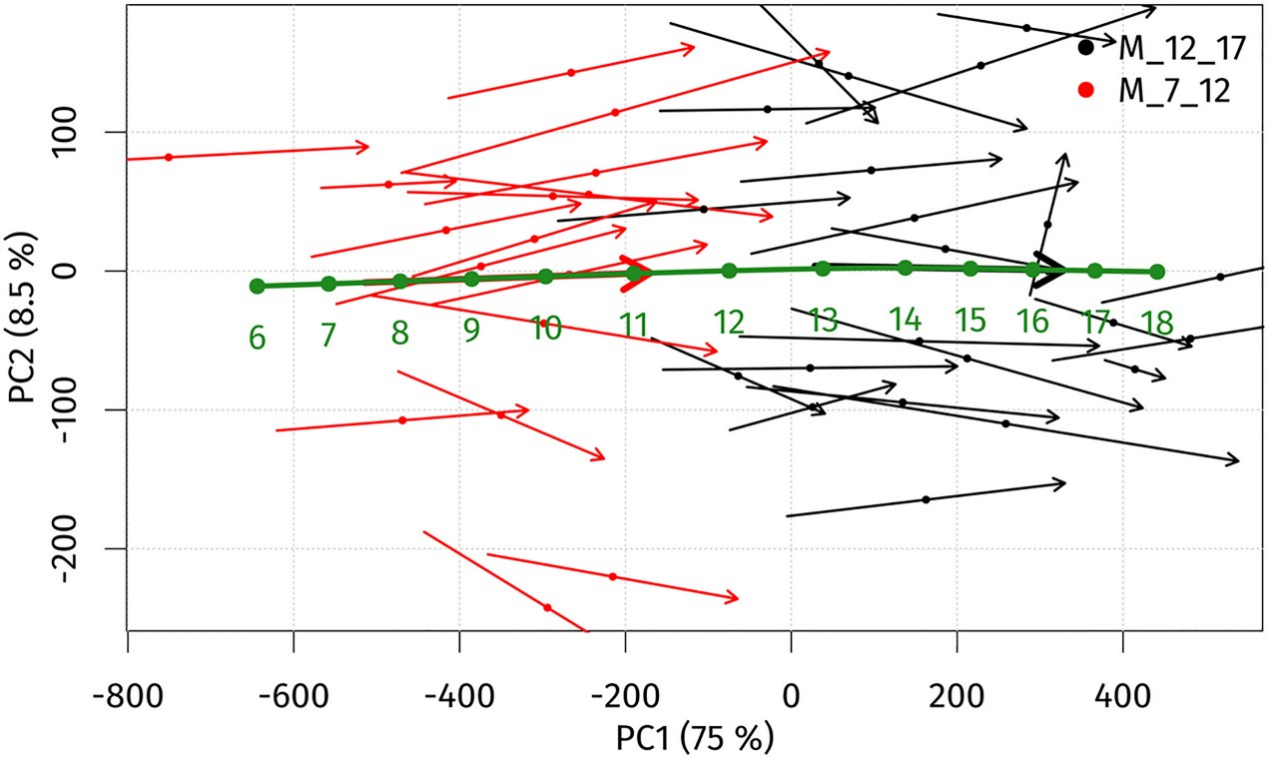
PCA scatter plot visualizing individual ageing trajectories (thin arrows), average ageing trajectories (thick arrows) and the global ageing trajectory (green arrow) of boys from 7 to 17 years of age in the space of the first (PC1) and second (PC2) principal components.

For boys, an identical PCA scatter plot with ageing trajectories was constructed (Fig 4). The first two principal components were responsible for over 83.5% of the sample’s variability. The first principal component (PC1) explained 79.2% of total variability and represents the facial ageing process. Towards positive values of PC1, the age of individual increased. PC2 explained 8.5% of total variability and was associated with facial concavity/convexity. In contrast to girls, the individual ageing trajectories of the younger group (from 7 to 12 years) and the older one (12 to 17 years) were similar in length. The global ageing trajectory showed the same annual changes up until 10 years of age. The most significant growth occurred from the age 10 to that of 14 years, with the maximum between 11 and 13 years of age. After the age of 14 years, annual growth changes slowly decreased.

**Fig 5:**
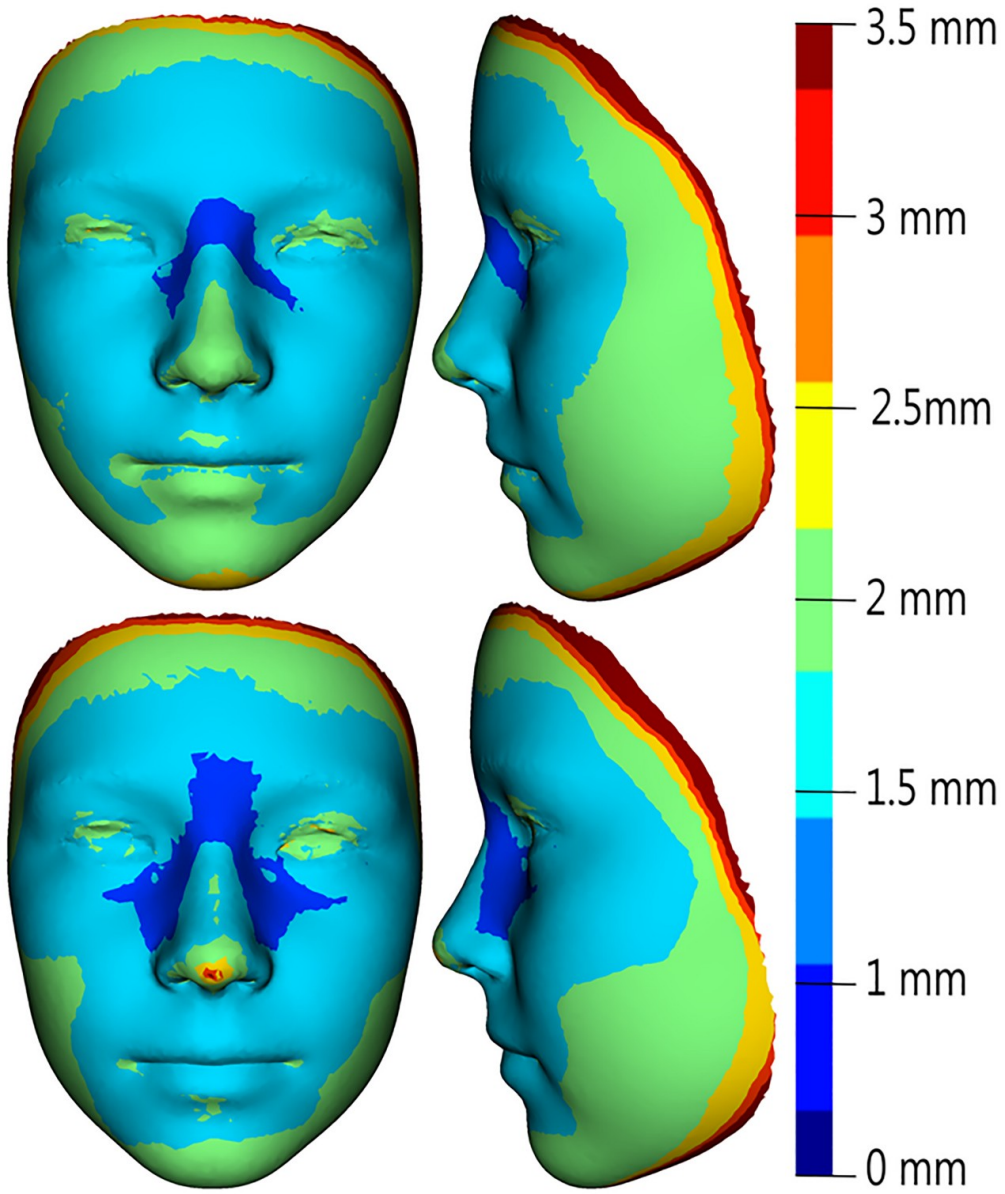
Visualization of mean error values obtained as the difference between the predicted and real facial surface in 12-year-old girls (upper row) and 17-year-old girls (lower row), as represented by facial colour-coded maps with a histogram (right).

**Fig 6:**
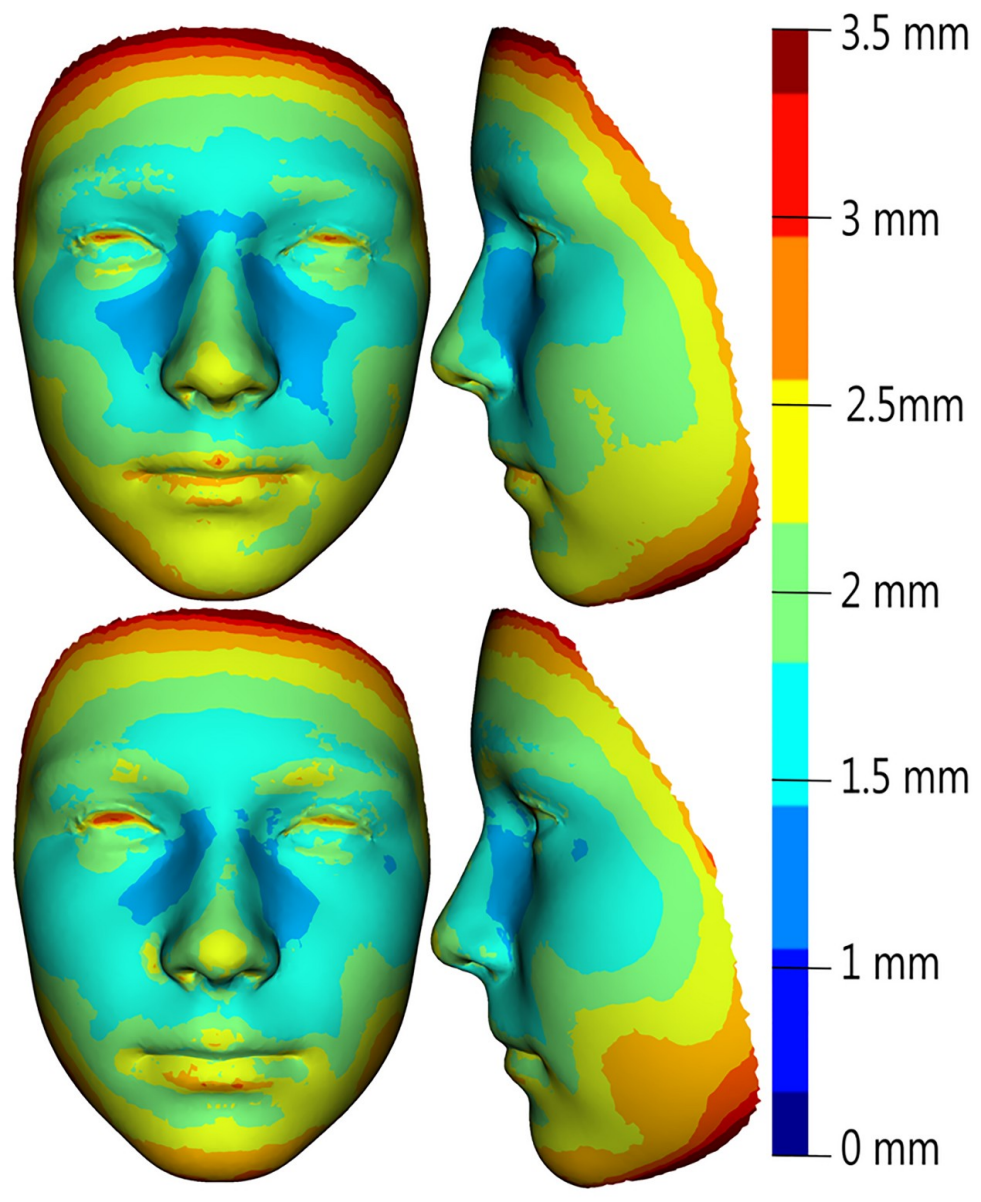
Visualization of mean error values obtained as the difference between the predicted and real facial surface in 12-year-old boys (upper row) and 17-years-old boys (lower row), as represented by facial colour-coded maps with a histogram (right).

The age progression (regression) model used the global ageing trajectories to predict facial morphology is shown in Figs 3 and 4. The model was tested by simulating ageing from 7 to 12 years and 12 to 17 years of age for both sexes separately. The mean error between real and predicted facial morphology at 12 and 17 years was visualized using superimposition and colour-coded maps. In girls, the mean error at 12 years of age was 1.81 mm, and at 17 years it reached 1.7 mm. The smallest deviations (of less than 1 mm) between real and predicted faces in both age categories were found in the glabellar area and the part around the nasal root (more pronounced in 17-year-olds—up to the lateral parts of nose). The major part of the forehead, together with the eyebrow ridges, the eyes and the whole orbital region, the nose (nasal ridge, tip, alae), the zygomatic region and the lips were areas with error values of less than 2 mm. The largest deviations (greater than 3 mm) were observed only in marginal parts of the whole face (Fig 5). In boys, the mean error between real and predicted faces at 12 years of age was 2.0 mm, and at 17 years of age it reached 1.94 mm. The areas with the smallest deviations (in boys set under 1.25 mm) were found in the glabellar area, lateral parts of the nose and part of the zygomatic region in 12 years old boys. In the oldest age category, the smallest error was observed just around the lateral parts of the nose. Areas with deviations of less than 2 mm were, in both age categories, situated in the central part of the face, with the exception of the eyelids, the nasal tip and a minor part of the eyebrow ridges in the older age category. The observed mean error was slightly greater in both age categories of boys compared with girls, but it was also situated only in marginal parts of the face (Fig 6).

For both age groups and sexes, our algorithm consistently improved the prediction compared to using the non-aged face (p<0.001 in all cases). In males the prediction was improved on average by 2.7 mm and 2.0 mm in 7 year-olds and 12 year-olds, respectively. Similarly, in females the prediction was improved by 3.0 mm and 1.1 mm, respectively.

**Fig 7:**
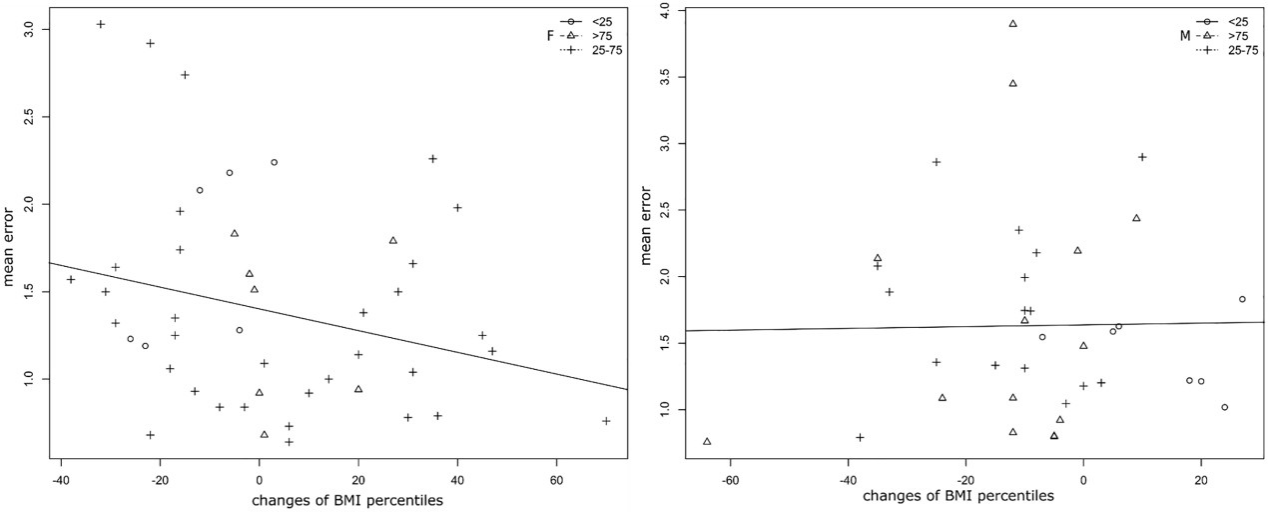
Scatter plot visualizing the relationship between changes of BMI percentiles (x-axis) and individual mean error (in mm) of an age progression (regression) model (y-axis) in three weight groups (<25,25–75 and >75 BMI percentiles) supplemented with a linear regression line for girls (left plot) and one for boys (right plot).

We compared changes of BMI percentiles throughout the observation period with each individual’s mean error of the age progression (regression) model. Using ANOVA, no statistical significant effect of changing BMI percentiles on the mean error was found in either of the sexes (p = 0.925 for boys, p = 0.0804 for girls). Nevertheless, according to the slope of the linear regression line and the distribution of the individuals (Fig 7), some differences between the two sexes were observed. In boys, changes of BMI percentiles during the observation period were smaller compared to girls, and no influence was apparent according to the slope of the regression line. In girls, the distribution of individuals indicated a shift to positive values of changes of BMI percentiles. The slope of the regression line indicates that the magnitude of the changes in BMI percentiles was inversely proportional to the mean error of the age-progression (regression) model. Nevertheless, the influence on the accuracy of the model was non-significant.

## Discussion

The understanding of changes in facial appearance caused by ageing is an essential point in several forensic disciplines, including age estimation, facial age recognition and age progression (age regression). Accurate methods are required in cases where individuals are undergoing criminal proceedings, are requesting asylum, in cases involving pedo-pornographic materials or the ever increasing number of missing children [40–42]. Effective facial age progression leading to the successful recovery of missing persons is important both for families and for law enforcement organizations. The age progression technique is also highly beneficial in different biomedical disciplines such as maxillofacial surgery or plastic surgery, especially when it comes to the planning of surgery or other medical procedures.

Numerous studies dealing with facial age progression have recently been published, mostly by computer vision scientists [13,43–46]. These authors usually assess the accuracy of their algorithms based on mathematical similarity between an age-progressed image and a target individual. Although this is well suited for their purposes, it is insufficient for determining the effectiveness of age-progression techniques in the real world, which requires assessment by human subjects [12]. Investigators of computational approaches are also limited by the number of high-resolution images depicting individuals over time, which are mostly two-dimensional, and by the cross-sectional nature of their approaches [10,25]. The absence of 3D facial models of missing persons that need to be age-progressed is another potential problem in real forensic scenarios, and methods of 3D face reconstruction from 2D photographs or images from video surveillance cameras have been intensively studied for many years [47–49]. In forensic practice, most age progressions are still produced by specialized artists, and the reliability of age progressions for the same individuals created by different artists can vary substantially [50]. It is important to develop more objective prediction methods that will be based on natural growth patterns. For this reason, the present study is based on a longitudinal evaluation, which allows to directly measure the mean error between real and predicted faces.

In a previous study [23] we found a strong influence of age on facial soft tissue morphology between 12 and 15 years of age. We reported differences in facial morphology as well as divergences in growth trajectories between the two sexes over the observation period. The process of facial development varies in different phases of the human life [51–56], so it is necessary to construct age progression models for specific periods.

In girls, we observed noticeable changes between 7 and 13 years of age, with the most significant changes occurring between 12 and 13 years of age. After the age of 13 years, the facial growth slowed down, and after 16 years of age it practically ceased. Bulygina et al. [51] also reported a significant decline in the rate of growth at approximately 13 years of age and a cessation of growth at about 15 years of age. Contrary to girls, facial changes in boys were evident over the entire observation period (up to the age of 17 years), but after the age of 16 years, growth changes were observed only in a minor part of the nose and chin. The most evident growth changes concerned the whole forehead region, the nose region and the mandible in both sexes. Similar results were observed in some previous studies [54,57–59]. Generally, facial changes were more intensive in boys compared to girls in most of the age categories except for the category between 10 and 11 years, in which the changes were the most noticeable in girls. The divergence in facial growth patterns was closely connected with the onset of puberty, which occurs about two years earlier in girls [28,51]. However, before puberty, males tend to have larger faces than females of the same age.

The different age-related facial surface changes were connected with the global ageing trajectories for both sexes. We observed very similar growth rates between the ages of 7 and 10 years in both sexes. Between 10 and 14 years of age, we observed increasing of growth velocity in both sexes, with maxima between 11 and 12 years in girls and between 11 and 13 years in boys. Similarly, Matthews et al. [59] reported different peaks in the rate of growth, with girls’ growth accelerating from approximately 11 years of age and boys’ accelerating from approximately age twelve. Bulygina et al. [51] similarly found that male and female growth trajectories are not always parallel but undergo some degree of divergence after the age of about 12 years. This divergence mainly concerns the change of shape; the magnitude of shape change per size change seems to be similar between the sexes in their study. Slightly different results were reported by Primozic et al. [60]. According to their study, the amount and velocity of facial growth in both sexes appears to be similar throughout the period of 6–12 years of age, irrespective of the presence of a pubertal growth spurt. Generally, the postnatal divergence of trajectories is an important aspect in developing adult shape differences [61].

Our age progression (regression) model achieved better results in facial prediction in both age categories of girls. However, the mean error between real and predicted faces was slightly lower in both older age categories (from 12 to 17 years of age) of girls and boys. By contrast, in our previous study [23], where we modelled the facial appearance from 12 to 15 years of age, the male age-progression model worked slightly better. In both our studies, the facial features with the smallest deviations were situated in the central part of the face. The mean error of our age-progression systems regardless of the age category did not exceed 2 mm. In the cross-sectional study [25], synthetically grown 3D faces were quantitatively compared with longitudinally collected images, and 85% of the faces were predicted correctly to within three millimetres.

The shape and configuration of internal facial features (i.e. the eyes, nose and mouth) are crucial criteria in facial recognition [62]. According to Tome et al. [63], the regions with the greatest discrimination power in facial recognition are the nose and forehead. Conversely, the external shape, which can conspicuously change during maturation, with weight gain or loss, or with changes in hairstyle, can affect identification accuracy [50] and may modify the representation of internal facial features in terms of face recognition [64].

Although the proportion of body fat affects facial shape [30–32], changes of BMI percentiles throughout the observation period had no significant influence on the accuracy of age our progression models for both sexes. Nevertheless, our present study indicates a slight influence on the accuracy of the model in girls. Although BMI is generally used as an accurate tool for estimating body fat mass in obese adolescents, differences in this index between thinner children can be largely attributable to fat-free mass [65], as BMI does not distinguish between body fat and muscle mass, which weighs more than fat. Nevertheless, body fat must be taken into consideration in studies of facial soft tissues [66].

The algorithm for facial prediction, which was based on longitudinal 3D data, was first presented in our previous study [23] a few years ago. The extension of the age range in the current study improved the accuracy of the age-progression model, which could become a very useful tool not only in several forensic disciplines, but also in different biomedical disciplines. The results show that our facial age progression framework produces considerably better ageing estimates than using a non-aged face. We assume, however, that each sex exhibits a common ageing pattern. Our framework does not take into account the initial form, changes in body composition or any other factors that would likely contribute to even more accurate predictions. Nevertheless, no ageing algorithm, regardless of what parameters it accounts for, can predict the true appearance of a subject with absolute accuracy. Therefore, all predictions should be considered tentative. Future investigations should be focused on the inclusion of facial texture to further improve the accuracy of age progression (regression) models, especially with regard to facial recognition.

## Supporting information

S1 File (ZIP)
